# *Shank3* Deficiency Results in a Reduction in GABAergic Postsynaptic Puncta in the Olfactory Brain Areas

**DOI:** 10.1007/s11064-023-04097-2

**Published:** 2024-01-06

**Authors:** Denisa Mihalj, Veronika Borbelyova, Zdeno Pirnik, Zuzana Bacova, Daniela Ostatnikova, Jan Bakos

**Affiliations:** 1 Institute of Experimental Endocrinology, Biomedical Research Center, Slovak Academy of Sciences, Dubravska cesta 9, Bratislava, 845 05 Slovakia; 2 Institute of Molecular Biomedicine, Faculty of Medicine, Comenius University, Bratislava, Slovakia; 3 Institute of Physiology, Faculty of Medicine, Comenius University, Bratislava, Slovakia; 4 Institute of Organic Chemistry and Biochemistry, Czech Academy of Sciences, Prague, Czech Republic

**Keywords:** Autism Spectrum Disorder, Olfactory System, *Shank3*, GABAergic Synapse

## Abstract

Dysfunctional sensory systems, including altered olfactory function, have recently been reported in patients with autism spectrum disorder (ASD). Disturbances in olfactory processing can potentially result from gamma-aminobutyric acid (GABA)ergic synaptic abnormalities. The specific molecular mechanism by which GABAergic transmission affects the olfactory system in ASD remains unclear. Therefore, the present study aimed to evaluate selected components of the GABAergic system in olfactory brain regions and primary olfactory neurons isolated from *Shank3*-deficient (^−/−^) mice, which are known for their autism-like behavioral phenotype. *Shank3* deficiency led to a significant reduction in GEPHYRIN/GABA_A_R colocalization in the piriform cortex and in primary neurons isolated from the olfactory bulb, while no change of cell morphology was observed. Gene expression analysis revealed a significant reduction in the mRNA levels of *GABA transporter 1* in the olfactory bulb and *Collybistin* in the frontal cortex of the *Shank3*^−/−^ mice compared to WT mice. A similar trend of reduction was observed in the expression of *Somatostatin* in the frontal cortex of *Shank3*^−/−^ mice. The analysis of the expression of other GABAergic neurotransmission markers did not yield statistically significant results. Overall, it appears that *Shank3* deficiency leads to changes in GABAergic synapses in the brain regions that are important for olfactory information processing, which may represent basis for understanding functional impairments in autism.

## Introduction

Dysfunctional sensory systems, including altered olfaction, have recently been suggested in patients with autism spectrum disorder (ASD) [1, 2]. A decrease in the amplitude and reliability of responses to odors has been reported in two mouse models of ASD [3]. Although many studies have focused on understanding the mechanisms of smell [4–8], the structural changes in neurite outgrowth, neuron shape, and neuronal markers associated with sensory alterations in ASD remain unclear. Olfactory nerve circuits include neurons of the olfactory bulb and their projections to the piriform cortex [8, 9]. It is also known that the olfactory bulb is characterized by the presence of many inhibitory interneurons that produce gamma-aminobutyric acid (GABA), and at the same time, different neuropeptides and neuromodulators, which undergo various neuroplastic changes during life [10, 11]. Although the classification of GABAergic neurons in the olfactory system can be based on various criteria such as their molecular markers, shape diversity, and functional properties, it is clear that interneurons make both local and long-range connections with neurons in other brain regions [12, 13]. GABAergic neurotransmission in the olfactory system involves the metabolism of GABA, transport of GABA by specific transporters, and binding of GABA to its receptors at the postsynaptic membrane [14, 15]. Dysfunction of GABAergic signaling at many levels is believed to be involved in the pathogenesis of ASD [16–18].

The role of potential changes in the olfactory system in ASD can be investigated using transgenic *Shank3*^−/−^ (*Shank3*-deficient) mice that are known to have ASD-like symptoms [19, 20]. In our previous studies, we confirmed behaviors relevant to ASD in this model, and at the same time, we characterized the presence of complex changes in neurotransmission markers, including GABA [21]. Although SHANK3 proteins are primarily components of glutamatergic synapses, several studies are currently analyzing the consequences of *Shank3* deficiency on GABAergic signaling in the context of ASD pathogenesis [20, 22]. One such marker is gephyrin, a scaffolding protein assembled in postsynaptic membranes that enables clustering and anchoring of GABA_A_ receptors (GABA_A_R) [23]. Its interaction with GABA_A_Rs facilitates the localization of these receptors, regulating the responsiveness of neurons to GABA signaling, thus modulating inhibitory neurotransmission in the brain. The evaluation of the colocalization of gephyrin and GABA_A_R allows the analysis of GABAergic synaptic puncta. To the best of our knowledge, no study using the *Shank3*^−/−^ mouse model of ASD has focused on GABAergic markers in the olfactory areas of the brain.

Therefore, in the present study, we hypothesized that *Shank3* deficiency results in (1) altered neurite outgrowth of neurons isolated from the olfactory bulb, (2) changed expression of inhibitory GABAergic markers in the olfactory bulb, and (3) changed inhibitory GABAergic synaptic puncta in neurons isolated from the olfactory bulb and in the olfactory region of the brain cortex. The olfactory bulb and piriform cortex were selected for analysis because of their fundamental roles in initial olfactory processing. The olfactory bulb serves as the primary site for organizing and relaying olfactory input, whereas the piriform cortex contributes to further processing and interpretation before transmitting information to higher brain regions for integration.

## Materials and Methods

### Experimental Animals

Pairs of heterozygous knockout B6.129-Shank3^tm2Gfng/J^ (*Shank3B*^*+/−*^) mice were obtained from the Jackson Laboratory (017688, JAX Laboratory, U.S.A.) and transported to the animal facility at the Faculty of Medicine of the Comenius University in Bratislava. Transgenic mouse pups were genotyped by PCR using DNA extracted from tail snips (approximately 3 mm) based on the Jackson Laboratory protocol. Only male *Shank3B*^*−/−*^ and *Shank3B*^*+/+*^ homozygous (wild type (WT) control) mice were used in this study. We utilized primary neuronal cell cultures from neonatal animals and tissue samples from two distinct age groups of animals to investigate potential changes during adolescence and adulthood. The mice were housed 3–4 per cages in animal facility under controlled laboratory conditions (12:12 h light/dark cycle with lights on 06:00–18:00 h, 22 ± 2 °C, 55 ± 10% humidity) with access to a standard pelleted diet and tap water *ad libitum*. All experimental procedures followed the ethical guidelines for animal experiments of the European Union Council (86/609/EEC) and was approved by the Ethical Committee of the Faculty of Medicine, Comenius University in Bratislava, Slovak Republic.

### Primary Cultures from Olfactory Bulb

Primary neuronal cells were isolated according to a protocol described by Reichova et al. [24]. Brains from the postnatal day 0 (P0; (3-4 pups/genotype) were dissected on ice-cold Hank’s Balanced Salt Solution –HBSS (137 mM NaCl; 5.4 mM KCl; 0.5 mM MgCl_2_ × 6 H_2_O; 0.4 mM MgCl_2_ × 6 H_2_O; 0.44 mM KH_2_PO_4_; 0.34 mM Na_2_HPO_4_ × 7 H_2_O; 1.25 mM CaCl_2_; 5.5 mM D-glucose) supplemented with 1% ATB and 0.3 M Hepes under a stereomicroscope to collect olfactory bulbs. The olfactory bulb tissues were dissociated by enzymatic treatment (HBSS, 0.1% Trypsin, 0.1 mg/ml DNAse I) for 20 min at 37 °C followed by 5 min incubation in RPMI medium (Sigma-Aldrich, Germany) containing 10% fetal bovine serum (HiClone, U.S.A.) at 37 °C. Resuspended cells were plated on 24-well plates containing glass coverslips pre-coated with 10 µg/ml Poly-D-lysine (Sigma-Aldrich, Germany) in RPMI medium containing 10% fetal bovine serum. After 3 h of cell plating, the medium was replaced with a selective growing medium, Neurobasal A (Gibco, U.S.A.), augmented with penicillin/streptomycin (100 U/ml), L-glutamine (2 mM) and B27 supplement (2% v/v; Invitrogen, U.S.A.). Primary neurons were then maintained at 37 °C in a humidified atmosphere (95% air and 5% CO_2_), and half of the growing medium was refreshed after 5 days in vitro (DIV5).

### Immunocytochemistry

At DIV10, the medium was removed, and primary cells from the olfactory bulb were fixed with 4% paraformaldehyde for 20 min at room temperature (RT). Coverslips were blocked in PBS containing 3% NGS and 0.1% Triton X-100 for 30 min at RT. Double immunofluorescence staining was performed by incubating the cells with rabbit anti-GEPHYRIN (PA5-29036, Invitrogen, 1:500 in PBS) and chicken anti-GABA_A_R (224 006, Synaptic Systems, 1:500 in PBS) for 2 h at RT. The coverslips were then washed in PBS and incubated with goat anti-rabbit (Alexa Fluor 555, A21428, Sigma-Aldrich, 1:500 in PBS) and goat anti-chicken (Alexa Fluor 647, A21449, Thermo Fisher Scientific, 1:500 in PBS) for 1 h at RT. Nuclei were stained with DAPI (Thermo Fisher, 1:1000 in PBS) for 1 min. After washing in PBS, the coverslips were mounted on slides using Fluoromount-G (Sigma-Aldrich, Germany).

### Tissue Processing

On P21 day, the first part of the mice (*n* = 9 per genotype) was sacrificed by decapitation, the brains were quickly removed from the skull, the olfactory bulbs were separated, deeply frozen in liquid nitrogen, and stored at -80 °C until RNA extraction. The second part of the adult mice (*n* = 6 per genotype) was deeply anesthetized with an intraperitoneal injection of sodium pentobarbital (Spofa, Czech Republic; 50 mg/kg), and immediately perfused transcardially with 4% paraformaldehyde in 0.1 M phosphate-buffered saline (PBS; pH 7.4). After perfusion, the brains were dissected and post-fixed by immersion in the same fixative overnight and then infiltrated with 20% sucrose in 0.1 M PBS for 48 h at 4 °C. Finally, the brains were frozen at -20 °C and then sectioned in the coronal plane at a thickness of 30 μm with the use of a cryostat (CM1950, Leica Microsystems GmbH, Germany). The free-floating sections were collected in cold PBS (4 °C), mounted on object slides and stored at -80 °C until further processing.

### Immunohistochemistry

To visualize the GEPHYRIN and GABA_A_R signal in the piriform cortex, selected sections (from bregma 1.18 mm to bregma 0.98 mm) in both *Shank3*^*−/−*^ and WT mice were subjected to double immunofluorescence. Briefly, the sections were triple-washed with cold PBS and then incubated for 1 h at RT with a blocking buffer composed of 0.1 M PBS, 3% normal donkey serum (NGS), 2% bovine serum albumin (BSA) and 0.1% Triton. After this, the sections were incubated overnight at 4 °C with primary antibodies diluted in a blocking buffer (PBS containing 1% NGS, 1% BSA, and 0.4% Triton). The primary antibodies were rabbit anti-GEPHYRIN (PA5-29036, Invitrogen, 1:500) and chicken anti-GABA_A_R (224 006, Synaptic Systems, 1:500). The next day, the sections were rinsed in cold PBS and incubated with secondary antibodies diluted in PBS for 1.5 h at RT. The secondary antibodies were goat anti-rabbit (Alexa Fluor 555, A21428, Sigma-Aldrich, 1:500) and goat anti-chicken (Alexa Fluor 647, A21449, Thermo Fisher Scientific, 1:500). After that, the sections were washed in PBS and stained with 4′,6-Diamidin-2-phenylindol (DAPI, Thermo Fisher, 1:1000 in PBS) for 15 min. Finally, the sections were mounted with Fluoromount-G (Sigma-Aldrich, Germany) and coverslipped.

### Image Acquisition

Images of fluorescent immunopositive signals were acquired using a confocal microscope (Nikon ECLIPSE Ti-E, A1R+, Netherlands) with a 40 × objective (numerical aperture 1.3; resolution 1024 × 1024 pixels) in 0.5 μm (brain sections) or 0.175 μm (primary neurons) steps. Double GEPHYRIN/GABA_A_R colocalization was quantified using ImageJ/Fiji software with ComDet Plugin [25]. Colocalized particles in the piriform cortex were independently detected in 274–284 regions of interest (ROI) per genotype, corresponding to 18 sections (6 parts of the bilateral piriform cortex/section, 3 ROI/each part of the piriform cortex) per genotype. Particle size was determined as 1 pixel (0,31 μm), with an intensity threshold of 3 pixels, and colocalization as a maximum distance of 1 pixel between particles. GEPHYRIN/GABA_A_R-positive clusters on dendrites were detected in 271–273 ROI per genotype, corresponding to 90 neuronal cells (3 ROI/cell) isolated from the olfactory bulbs per genotype. MAP2 (Microtubule-Associated Protein 2) positive cells were considered as neurons. Three coverslips per animal and at least 10 areas of interest per coverslip were evaluated. Particle size was determined as 1 pixel (0.31 μm), with an intensity threshold of 20 pixels, and colocalization as a maximum distance of 1 pixel between particles. Quantification of the longest neurite length was performed using the Fiji/ImageJ software on 98 neuronal cells isolated from the olfactory bulbs per genotype. The length of the longest neurite was quantified from the edge of the nucleus to the apical end. Based on the length of the longest neurite, the neurons were divided into 4 categories (up to 50 μm, 50–100 μm, 100–150 μm, and over 150 μm) for each genotype and analyzed the number of neurons with the longest neurite expressed as a percentage of the total measured neurons.

### Quantitative Real Time PCR Analysis

Total RNA was extracted from olfactory bulb tissues using the phenol-chloroform method with TRI reagent (Molecular Research Center, Germany). The concentration and purity of RNA were determined using a Nanodrop spectrophotometer (Thermo Fisher Scientific, U.S.A.). First-strand cDNA was synthesized using a High-Capacity cDNA Reverse Transcription Kit (Thermo Fisher Scientific, U.S.A.) according to the manufacturer’s protocol. Analysis of the expression levels of selected genes was performed using PCR primers designed with Primer-Blast and Power SYBR® Green PCR Master Mix (Thermo Fisher Scientific, U.S.A.), according to the manufacturer’s instructions (sequences of specific primers in Table 1). Quantitative real-time polymerase chain reaction (qRT-PCR) was performed using a QuantStudio5 Real-Time PCR System (Thermo Fisher Scientific, U.S.A.). The thermocycling conditions were as follows: 50 °C for 2 min, 95 °C for 10 min, 50 cycles at 95 °C for 15 s, and 60 °C for 1 min. The relative differences in gene expression between the control and experimental groups were calculated using threshold cycle (CT) values that were first normalized to the housekeeping gene 18s, which served as endogenous controls in the same sample, and then relative to a control CT value using the 2^−ΔΔCT^ method [26].

**Table 1:** List of primer sequences used in this study. forward (Fw), reverse (Rv); 4-aminobutyrate aminotransferase (*Abat*), Collybistin (*Arhgef9*), GABA_A_R receptor-associated protein-like (*Gabarapl*), Gamma-aminobutyric acid receptor subunit alpha (*Gabra*), Glutamate decarboxylase (*Gad*), GABA transporter (*Gat*), The vesicular GABA transporter (*Vgat*), ribosomal protein 18 S (*18s*)

Name		Primers	Gene Bank	References
***Abat***	Fw: GGACTTCCGTCTTCATGAGTG Rv: ACCTCCACCTCTTCATACCT		NM_172961.3	[27]
***Arhgef9***	Fw: CAAGGAAACGGAAGAAGTGC Rv: GGGCAGAGTTGACACCTTTC		NM_001290385.1	own design
***Gabarapl1***	Fw: CATCGTGGAGAAGGCTCCTA Rv: ATACAGCTGGCCCATGGTAG		NM_020590.4	[28]
***Gabarapl2***	Fw: TCACTGTGGCTCAGTTCATG Rv: TAGTTAGGCTGGACTGTGGG		NM_026693.5	[29]
***Gabra1***	Fw: CTCTCCCACACTTTTCTCCC Rv: CCGACAGTGTGCTCAGAATG		NM_010250	[30]
***Gabra2***	Fw: AGATTCAAAGCCACTGGAGG Rv: CCAGCACCAACCTGACTG		NM_008066.4	[30]
***Gad65***	Fw: GACCAATCTCTGTGACTCGCTTAG Rv: CTGGTCAGTGTATCGGAGGTCTT		NM_008078.2	[31]
***Gad67***	Fw: CATGGTCATCTCAAACCCTGC Rv: CGAGGCGTTCGATTTCTTCA		NM_008077.5	[31]
***Gat1***	Fw: TAACAACAACAGCCCATCCA Rv: GGAGTAACCCTGCTCCATGA		NM_178703.4	[32]
***Gat3***	Fw: CTATGATGCCCCTCTCTCCAC Rv: CTGTCACAAGACTCTCCACG		NM_172890.3	[33]
***Gephyrin***	Fw: GACAGAGCAGTACGTGGAACTTCA Rv: GTCACCATCATAGCCGTCCAA		NM_172952	[31]
***Parvalbumin***	Fw: TGCTCATCCAAGTTGCAGG		NM_013645.4	[30]
	Rv: GCCACTTTTGTCTTTGTCCAG			
***Somatostatin***	Fw: AGGACGAGATGAGGCTGG		NM_009215	[30]
	Rv: CAGGAGTTAAGGAAGAGATATGGG			
***Vgat***	Fw: GGGCTGGAACGTGACAAA		NM_001421187.1	[34]
	Rv: GGAGGATGGCGTAGGGTAG			
***18s***	Fw: CGCCGCTAGAGGTGAAATTC		NM_001081383.2	[35]
	Rv: TTGGCAAATGCTTTCGCTC			

### Statistical Analysis

Statistical analysis was performed using GraphPad Prism, version 8.0 (GraphPad Software Inc., CA, U.S.A.) and Excel XLSTAT plugin (XLSTAT-Premium, Addinsoft, New York, U.S.A.). The data were first tested for normal distribution using the Shapiro–Wilk test. If the data were normally distributed, the two-group means were analyzed using an unpaired two-tailed Student’s t-test. If the data distribution was not normal, the non-parametric Mann-Whitney or Kolmogorov-Smirnov test was used. Figures were generated using SigmaPlot 10.0.1 (Systat Software Inc., CA, U.S.A.). Box plots were used to report the distribution of colocalized particles and contain median, interquartile range (IQR) between the lower quartile (Q1, 25%) and the upper quartile (Q3, 75%). Data points that fell below Q1–1.5 * IQR or above Q3 + 1.5 * IQR were considered outliers and were therefore excluded from the analysis. All other data were expressed as mean ± standard deviation (SD). Statistical significance was set at *p* < 0.05.

## Results

### Primary Neurons Isolated from the Olfactory Bulb of *Shank3*-deficient Mice Have Reduced GABAergic Puncta

First, we determined whether *Shank3* deficiency could affect neuronal morphogenesis by analyzing the length of the longest neurites. Based on the length of the longest neurite, we divided the neurons into 4 categories (up to 50 μm, 50–100 μm, 100–150 μm, and over 150 μm) for each genotype and analyzed the number of neurons with the longest neurite expressed as a percentage of the total measured neurons. Overall, we did not find changes in the longest neurites of neurons isolated from the olfactory bulb of *Shank3*^−/−^ mice compared to neurons from control WT mice (Kolmogorov-Smirnov test; D = 0.1398; *p* = 0.3236), and the percentage of neurons with the longest neurites did not significantly differ in any length category.

**Fig. 1:**
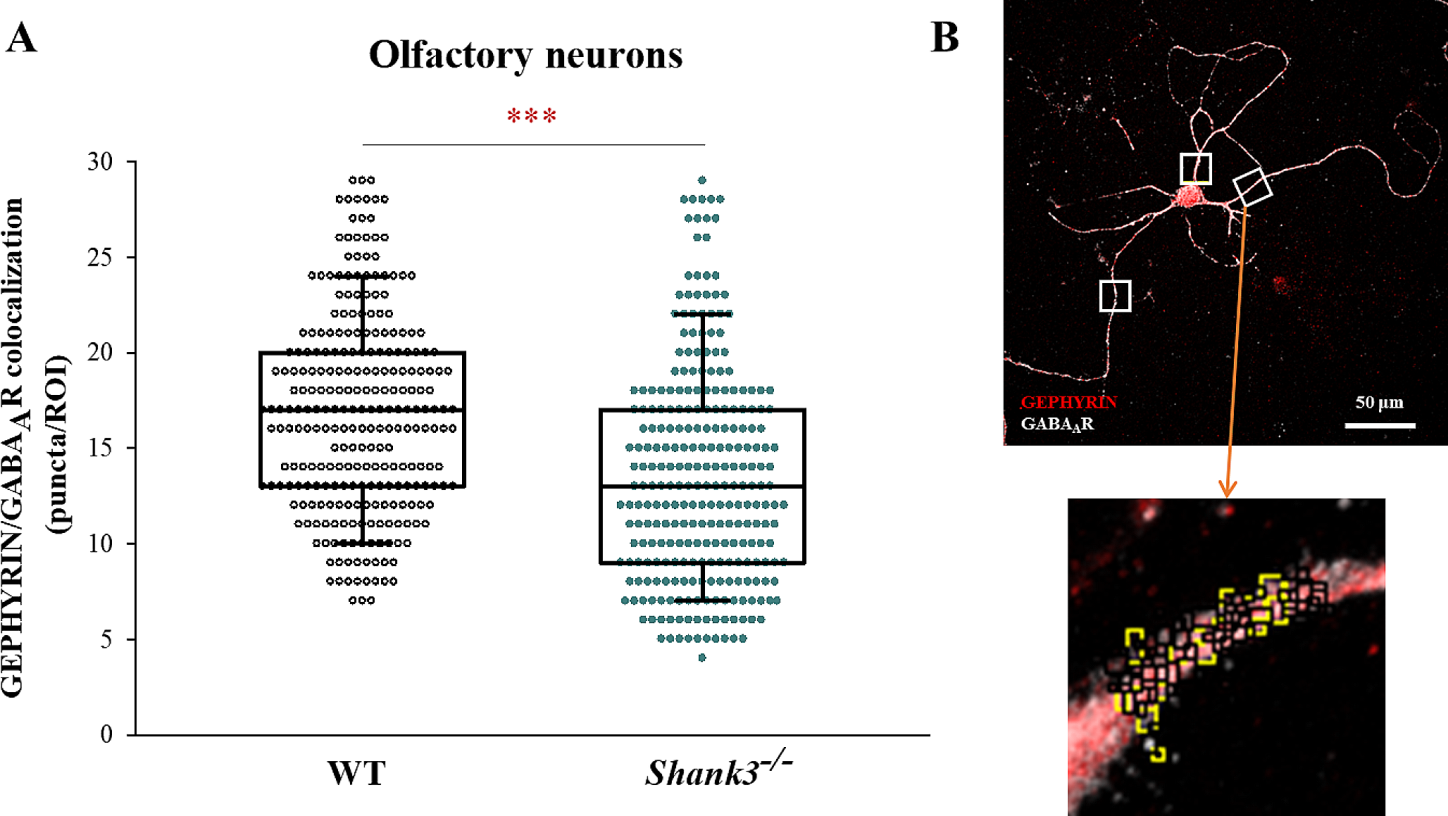
The number of GEFHYRIN/GABA_A_R-positive clusters in the dendritic regions of primary olfactory neurons in WT and Shank3^−/−^ mice. **A**) Statistical significance between medians of colocalized particles (ROI_(WT)_ = 271, ROI_(KO)_ = 273) was determined using the Kolmogorov-Smirnov non-parametric test (***p < 0.001). The boxes enclose 50% of the data and represent the median and the 25% and 75% quartiles, respectively. Dots represent individual ROI values. Number of mice: N_(WT)_ = 3 pups; N_(KO)_ = 4 pups. **B**) Schematic representation of confocal microscopy images of primary olfactory bulb neuron (40x magnification) showing GEPHYRIN and GABA_A_R puncta (black squares), as well as their colocalization (yellow squares). Scale bars represent 50 μm; white boxes represent ROI (20 × 20 μm)

To investigate whether olfactory neurons isolated from *Shank3*^−/−^ mice differ in subtle synaptic changes, we evaluated GABAergic synaptic puncta. Statistical analysis revealed a significant reduction (Kolmogorov–Smirnov test; D = 0.257; *p* < 0.001) in GEPHYRIN/GABA_A_R colocalization in primary neurons isolated from the olfactory bulb of *Shank3*^−/−^ mice compared to WT control neurons (Fig. 1A).

### The Olfactory Areas of The Brain of *Shank3*^*-/-*^ Mice Have Slight Changes in Selected GABAergic Markers, and at The Same Time, Markedly Reduced GABAergic Clusters

Furthermore, we analyzed changes in the entire spectrum of GABAergic markers in the tissues of the olfactory bulb and frontal cortex (Table 2). Similar to the olfactory bulb, the frontal cortex also includes local circuits of GABAergic interneurons that are important for the regulation of cognitive and social functions in ASD [36]. For the analysis of GABAergic interneuron markers, the major transcripts for somatostatin and parvalbumin were selected. Gene expression analysis showed a significant reduction in the mRNA levels of GABA transporter 1 (*Gat1*) in the olfactory bulb of *Shank3*^***−/−***^ mice compared to WT control (Mann-Whitney test; U = 47; *p* = 0.029). Also, expression levels of *Collybistin* were significantly reduced in the frontal cortex of *Shank3*^***−/−***^ mice (Mann-Whitney test; U = 57; *p* = 0.046). *Somatostatin* expression levels showed a trend toward reduction in the frontal cortex of *Shank3*^***−/−***^ mice, but this was not statistically significant (*p* = 0.059; df = 13; t = 2.16). The analysis of the expression of other GABAergic neurotransmission markers did not yield statistically significant results.

**Table 2:** Changes in the transcript levels of selected GABAergic synapse markers in the olfactory bulb (OB) and frontal cortex (FC) of WT and *Shank3*^*−/−*^ mice. Values show relative mRNA levels normalized to *18s* transcript. qRT-PCR was calculated by the 2^−ΔΔCt^ Livak method [26]. Represented are means ± SD (*n* = 6–9/genotype). Statistical significance between values was determined using the two-tailed Student’s t-test of Mann-Whitney non-parametric test (*p < 0.05). 4-aminobutyrate aminotransferase (*Abat*), GABA_A_R receptor-associated protein-like (*Gabarapl*), Gamma-aminobutyric acid receptor subunit alpha (*Gabra*), Glutamate decarboxylase (*Gad*), GABA transporter (*Gat*), The vesicular GABA transporter (*Vgat*)

Gene	OB mRNA levels		FC mRNA levels	
	**WT**	***Shank3*** ^***−/−***^	**WT**	***Shank3*** ^***−/−***^
***Somatostatin***	1,19 ± 0,84	1,62 ± 0,52	1,01 ± 0,16	**0,87 ± 0,08**
***Parvalbumin***	1,11 ± 0,54	0,90 ± 0,39	1,02 ± 0,39	0,89 ± 0,39
***Abat***	1,06 ± 0,22	0,91 ± 0,40	1,06 ± 0,60	0,94 ± 0,18
***Gad65***	0,92 ± 0,27	1,04 ± 0,29	1,11 ± 0,44	1,41 ± 1,00
***Gad67***	1,02 ± 0,26	0,86 ± 0,40	1,07 ± 0,34	0,87 ± 0,37
***Vgat***	1,31 ± 1,22	1,00 ± 0,52	0,72 ± 0,21	0,67 ± 0,24
***Gat1***	1,12 ± 0,64	0,57 ± 0,32*	1,04 ± 0,45	0,78 ± 0,30
***Gat3***	0,91 ± 0,21	1,12 ± 0,29	1,18 ± 0,63	1,20 ± 0,52
***Gephyrin***	0,87 ± 0,11	0,75 ± 0,16	0,87 ± 0,07	0,89 ± 0,34
***Collybistin***	1,05 ± 0,36	0,76 ± 0,32	1,14 ± 0,70	0,65 ± 0,23*
***Gabarapl1***	1,00 ± 0,11	0,88 ± 0,19	1,00 ± 0,10	1,06 ± 0,34
***Gabarapl2***	1,07 ± 0,34	0,89 ± 0,28	1,04 ± 0,35	0,88 ± 0,24
***Gabra1***	1,01 ± 0,20	1,02 ± 0,22	0,75 ± 0,10	0,67 ± 0,17
***Gabra2***	1,10 ± 0,50	0,82 ± 0,36	0,69 ± 0,16	0,60 ± 0,10

**Fig. 2:**
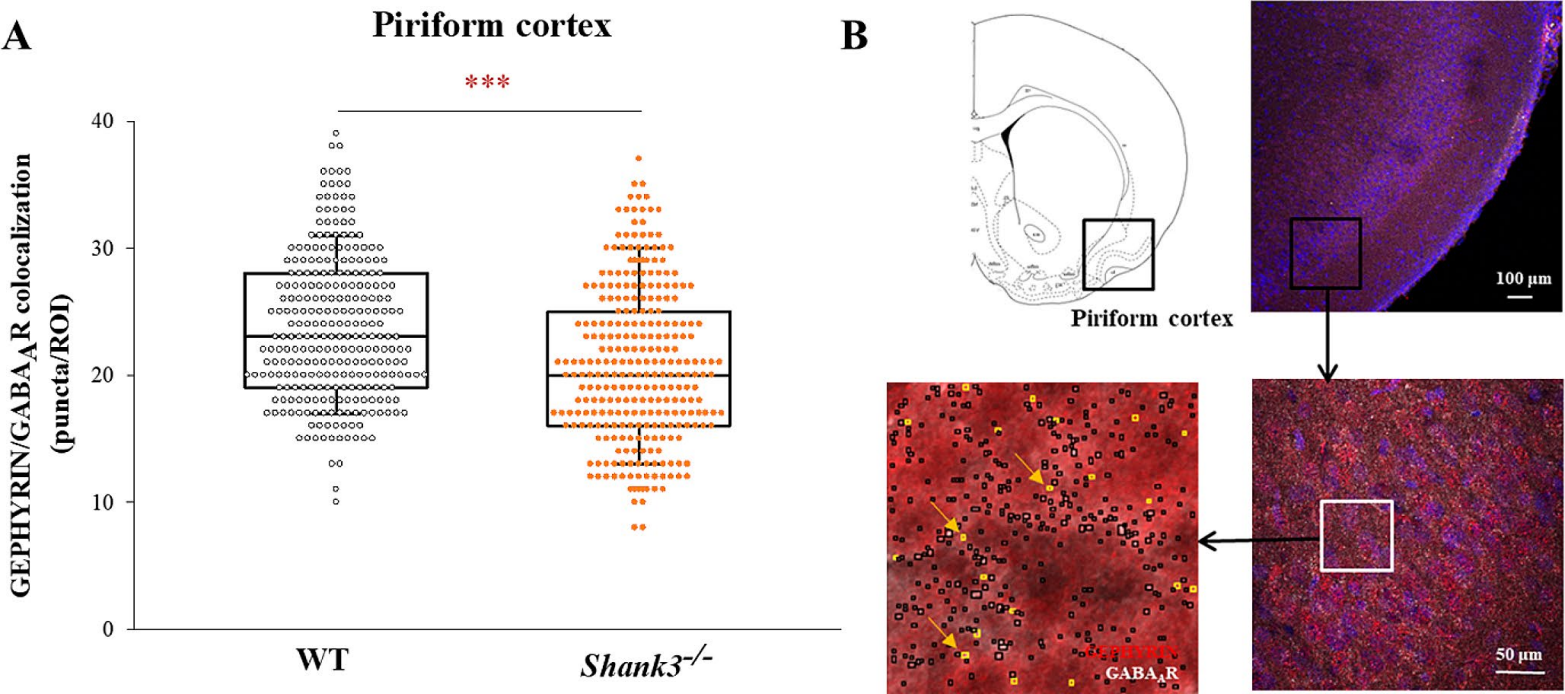
Effect of *Shank3* deficiency on colocalization between GEPHYRIN/GABA_A_R particles in the piriform cortex. **A**) Statistical significance between medians of colocalized particles (ROI_(WT)_ = 274, ROI_(KO)_ = 284) was determined using the Kolmogorov-Smirnov nonparametric test (***p < 0.001). The boxes enclose 50% of the data and represent the median and the 25% and 75% quartiles, respectively. Dots represent individual ROI values. Number of mice: N_(WT)_ = 6; N_(KO)_ = 6. (**B**) Schematic representation of the chosen region (piriform cortex) and confocal microscopy images (10x and 40x magnification) showing GEPHYRIN and GABA_A_R puncta (black squares), as well as their colocalization (yellow squares) in coronal brain slices. Scale bars represent 100 μm or 50 μm; white box represents ROI (60 × 60 μm)

To determine whether the change in GABA transport in the olfactory system in *Shank3*^*−/−*^ mice can have consequences on olfactory projection areas, we evaluated postsynaptic GABAergic clusters in the piriform cortex. We found a significant decrease in the colocalization of GEPHYRIN/GABA_A_R in the piriform cortex (Kolmogorov–Smirnov test; D = 0.199; *p* < 0.001) (Fig. 2A).

## Discussion

Understanding the structural differences in the sensory areas of the brain of *Shank3*^*−/−*^ mice is an important prerequisite for explaining previously described and partially known functional electrophysiological and behavioral deficits in animal models of ASD [3, 21, 37]. In this study, we found a significant reduction in specific GABAergic puncta in primary olfactory neurons in vitro, as well as in the piriform cortex in vivo. These changes correspond to a reduction in the expression of the selected GABAergic markers.

Our hypothesis that neurons isolated from the olfactory bulb of *Shank3*^*−/−*^ mice would differ in their morphology was not confirmed. Another study, using a different model utilizing the CRISPR/Cas9 editing system for the *Shank3* gene, found reduced neurite count and neurite length of neurons from inducible pluripotent stem cells reprogrammed and derived directly from patients with ASD [38]. Other authors have described different morphogenetic abnormalities (i.e., more extensively branched neurites) in *Shank3* knockout neurons derived from human embryonic stem cell lines [39]. Although it has been suggested that olfactory axons display defective pathfinding in animal models of ASD [40], we did not observe any alterations in the neurite outgrowth of olfactory neurons. However, it cannot be ruled out that a specific population of olfactory neurons could show certain changes in the growth of neurites or their shape. Modification in genes related to the pathogenesis of ASD affects the growth, shape and arborization of different subtypes of neurons [24, 41, 42]. Another possibility that comes to play is that the enlarged brain volume observed in some individuals with ASD may be associated with altered neurite outgrowth and increased dendritic arborization [43, 44]. This potential overgrowth of neural connections and increased complexity in neuronal branching could contribute to the larger brain volume observed in certain cases of ASD.

Our next working hypothesis was that neurons in *Shank3*^*−/−*^ mice would differ in subtle differences in their GABAergic synaptic connections. We found a marked reduction in GABAergic puncta in isolated primary neurons as well as in immunohistochemical sections of the piriform cortex. This finding is important because it was consistently seen in the early development stages and in samples from adult animals. Although our study did not distinguish subtypes of inhibitory interneurons, the results clearly indicate that *Shank3* deficiency leads to alterations in both local and long-range GABAergic connections. Moreover, we observed a significant decrease in the gene expression of selected GABAergic markers (*Gat1* and *Collybistin*) in the brain of *Shank3*^*−/−*^ mice. More pronounced changes in the expression of GABAergic synapse components could be masked by homogenization of the whole tissue and the technical limits of the PCR methodology. Although not directly in the olfactory parts of the brain, other studies in *Shank3*^*−/−*^ mice showed a reduction in the mRNA levels of GABA_A_R subunits or *Parvalbumin* in the prefrontal cortex or hippocampus [22, 45].

The concept of deficits in GABAergic signaling in ASD is not entirely new, and many data have been collected to date (e.g., [46, 47]). Our findings extend this concept to the reduction of GABA markers in the olfactory system caused by *Shank3* deficiency. It is possible that impaired GABA_A_R-mediated synaptic transmission contributes to functional changes in smell, which should be further tested in *Shank3*^*−/−*^ mice or other ASD animal models. Although we did not perform olfactory tests in this study, decreased interest in unfamiliar social olfactory cues was found in another ASD mouse model [48]. One possible explanation proposed by these authors is the impaired GABA_A_R trafficking. Alterations in the spatial arrangement of GABAergic receptors are associated with abnormal connectivity, particularly in relation to the processing of sensory information in ASD [49]. Therefore, changes in GABAergic neurotransmission were considered in the present study.

The limitations of our study include the fact that only selected areas of the olfactory system were investigated, and it is necessary to mention that further studies should focus on higher-order centers of olfactory information processing, that is, the anterior olfactory nucleus, olfactory tubercle, cortical nucleus of the amygdala, or entorhinal cortex. A second limitation is that we could not directly compare individual developmental stages, since we did not evaluate all their parameters. A third limitation is the lack of behavioral olfactory sensitivity testing, which could be functionally altered, however, this assumption in connection with inhibitory neurotransmission requires further testing.

In conclusion, our findings on the reduction of GABAergic synapse components are important for clarifying the pathological implications of *Shank3* deficiency. In a wider context, the results of the present study contribute to the knowledge of GABArgic abnormalities in ASD-like conditions, which can manifest as impairments in the olfactory system.

## Data Availability

The data that support the findings of this study are available from the corresponding author upon reasonable request.
